# Association of Hyperbaric Oxygen Therapy with Platelet Reactivity in Patients with Advanced Peripheral Arterial Disease: A Prospective Observational Study

**DOI:** 10.3390/jcm15103723

**Published:** 2026-05-12

**Authors:** Dragan Knezevic, Vladimir Zivkovic, Vladimir Jakovljevic, Nikola Mirkovic, Milena Ilic, Marija Andjelkovic, Jelena Mijajlovic, Vladimir Fisenko, Goran Balovic, Djordje Kolak

**Affiliations:** 1 Department of Surgery, Faculty of Medical Sciences, University of Kragujevac, 34000 Kragujevac, Serbia; drnikolamirkovic@gmail.com (N.M.); gbalovic@gmail.com (G.B.); djordje.kolak@gmail.com (D.K.); 2 Clinical Center Kragujevac, 34000 Kragujevac, Serbiamarijabcd@gmail.com (M.A.); drjelena74@gmail.com (J.M.); 3 Department of Physiology, Faculty of Medical Sciences, University of Kragujevac, 34000 Kragujevac, Serbiadrvladakgbg@yahoo.com (V.J.); 4 Center of Excellence for Redox Balance Research in Cardiovascular and Metabolic Disorders, Faculty of Medical Sciences, University of Kragujevac, 34000 Kragujevac, Serbia; 5 Department of Pharmacology, Sechenov University, 119991 Moscow, Russia; vpfisenko@mail.ru; 6 Department of Human Pathology, I.M. Sechenov First Moscow State Medical University, 119991 Moscow, Russia; 7 Department of Pathology, Faculty of Medical Sciences, University of Kragujevac, 34000 Kragujevac, Serbia; 8 Department of Medical Biochemistry, Faculty of Medical Sciences, University of Kragujevac, 34000 Kragujevac, Serbia

**Keywords:** peripheral arterial occlusive disease, hyperbaric oxygen therapy, platelet aggregation, Multiplate, platelet reactivity, chronic limb-threatening ischemia, ischemic ulcer

## Abstract

**Objective:** Peripheral arterial occlusive disease (PAOD) is characterized by impaired tissue perfusion, chronic ischemia, and increased platelet reactivity. Hyperbaric oxygen therapy (HBOT) is used as adjunctive treatment in advanced PAOD, but its effect on platelet function remains insufficiently studied. This study examined the association between HBOT and platelet aggregation. **Methods:** This prospective observational study included 90 patients with Fontaine stage IV PAOD and chronic ulceration, assigned to an HBOT group (*n* = 60) or waiting-list control group (*n* = 30). Patients were predominantly male; mean age was 66.82 ± 9.42 years in the study group and 63.00 ± 8.31 years in controls, and diabetes mellitus was present in 55.0% and 63.3%, respectively. Prior revascularization included open surgery in 33.3% and 30.0%, endovascular treatment in 36.7% and 43.3%, and no option for revascularization in 30.0% and 26.7%, respectively. HBOT was administered over 4 weeks (20 sessions, 2.0–2.5 ATA). Platelet aggregation was measured by impedance aggregometry using arachidonic-acid-induced aggregation (ASPI), adenosine-diphosphate-induced aggregation (ADP), and thrombin-receptor-activating peptide-induced aggregation (TRAP) agonists. Changes were analyzed using generalized estimating equation models adjusted for antiplatelet therapy, diabetes mellitus, smoking, and C-reactive protein (CRP). **Results:** Significant group × time interactions were observed for all platelet activation pathways, indicating greater reductions in the HBOT group than controls: ASPI (β = −290.5; *p* < 0.001), ADP (β = −243.6; *p* < 0.001), and TRAP (β = −330.9; *p* < 0.001). No significant change was observed in controls. HBOT was associated with reduced pain intensity, while CRP and platelet-to-lymphocyte ratio (PLR) remained stable. Ulcer size showed no significant change after 4 weeks. **Conclusions:** In patients with PAOD, HBOT was associated with reduced platelet reactivity independent of antiplatelet therapy. Further randomized studies are needed to determine its clinical significance.

## 1. Introduction

Peripheral arterial occlusive disease (PAOD) is primarily caused by atherosclerosis. It mainly affects the arteries of the lower extremities, leading to progressive luminal narrowing, possible occlusion, and distal tissue ischemia [1]. It affects over 200 million individuals worldwide, with annual healthcare costs in the United States of approximately USD 6.3 billion, and its prevalence increases with age and cardiovascular risk factors such as hypertension, diabetes mellitus, dyslipidemia, and tobacco use [1]. Its pathophysiology involves endothelial dysfunction, oxidative stress, chronic inflammation, coagulation activation, and a prothrombotic state, resulting in plaque formation. Clinically, PAOD presents with intermittent claudication and may progress to critical limb ischemia [2].

Platelets play a central role in atherosclerosis, with increased reactivity and aggregation in peripheral vascular disease, as demonstrated by light transmission aggregometry. Markers of platelet activation, including P-selectin expression, have been associated with ischemic outcomes in patients with peripheral artery disease, while high on-treatment platelet reactivity may persist despite antiplatelet therapy in a substantial proportion of patients [3].

Recent studies combining functional platelet testing with transcriptomic profiling have identified genetic signatures of platelet hyperreactivity in PAOD, with higher Platelet Reactivity Expression Score (PRESS) values predicting increased risk of major cardiovascular events after revascularization [4]. Chronic ischemia and reduced oxygen availability further promote a prothrombotic state characterized by enhanced platelet reactivity and aggregation [5,6].

Diabetes mellitus is a major risk factor for PAOD, with peripheral vascular damage often present at diagnosis, particularly in low- and middle-income countries due to delayed detection [7,8]. It also affects platelet function, being associated with increased receptor expression, enhanced aggregation, and reduced responsiveness to antiplatelet therapy, thereby increasing thrombotic risk in patients with PAOD [9].

Management of PAOD includes risk-factor modification, pharmacologic therapy (statins, antihypertensive and antiplatelet agents), and endovascular or surgical revascularization. However, persistent ulcerative lesions may occur despite optimal treatment, particularly in patients with diabetes, in whom diabetic foot complications often progress to major amputation [10]. These limitations highlight the need for additional therapies targeting microcirculation, tissue oxygenation, and the prothrombotic state.

Hyperbaric oxygen therapy (HBOT) involves inhalation of 100% oxygen under increased pressure, enhancing plasma-dissolved oxygen and delivery to hypoxic tissues [10]. In addition to improved oxygenation, HBOT modulates endothelial function, oxidative stress, inflammation, and hemostatic pathways, and is widely used as an adjunctive treatment for non-healing wounds in diabetes and/or peripheral arterial disease to promote healing and reduce amputation risk [10].

Evidence suggests that HBOT may improve wound healing and reduce amputation rates in these patients, although results are heterogeneous due to differences in study design and protocols. Beyond tissue repair, HBOT improves microcirculation and modulates inflammatory and angiogenic biomarkers [10,11]. It may also influence hemorheological properties and platelet function, including reduced blood viscosity, improved erythrocyte deformability, and modulation of platelet aggregation, potentially reducing hypercoagulability and improving microcirculatory flow.

Therefore, this study aimed to evaluate whether HBOT is associated with changes in platelet aggregation in patients with PAOD.

## 2. Materials and Methods

### 2.1. Study Design

This prospective longitudinal observational study evaluated the association between hyperbaric oxygen therapy (HBOT) and platelet reactivity in patients with advanced peripheral arterial disease (Fontaine stage IV). The primary outcome was the change in platelet aggregation assessed by the Multiplate^®^ analyzer (Roche Diagnostics GmbH, Mannheim, Germany) arachidonic-acid-induced aggregation (ASPI) (AUC) from baseline to 4 weeks, comparing the HBOT and waiting-list control groups. Secondary outcomes included changes in adenosine-diphosphate-induced aggregation (ADP), and thrombin-receptor-activating peptide-induced aggregation (TRAP) agonists (AUC), inflammatory markers C-reactive protein (CRP), platelet-to-lymphocyte ratio (PLR), and pain intensity assessed using the visual analogue scale (VAS) over 4 weeks. This study was reported in accordance with the STROBE (Strengthening the Reporting of Observational Studies in Epidemiology) guidelines. The study was conducted in accordance with the Declaration of Helsinki and approved by the Ethics Committee of the Faculty of Medical Sciences, University of Kragujevac (Approval No. 01-1743/2). Written informed consent was obtained from all participants.

Patients with chronic lower-extremity ulcerations due to PAOD, regardless of diabetes status, were treated with HBOT at the Center for Hyperbaric Medicine, Faculty of Medical Sciences, University of Kragujevac, from September 2024 to June 2025. Chronic ulceration was defined as a wound persisting for more than four weeks [12].

Eligible patients were consecutively enrolled according to predefined inclusion and exclusion criteria in order to reduce selection bias and reflect routine clinical practice. Randomization was not performed because access to HBOT depended on chamber capacity and scheduling availability within routine care. Patients who were able to start HBOT immediately were assigned to the HBOT group, whereas those scheduled to start HBOT after the predefined 4-week observation period served as waiting-list controls during that interval. Allocation was therefore determined by treatment availability rather than investigator preference or baseline clinical characteristics, including platelet reactivity, ulcer size, pain severity, and diabetes status.

### 2.2. Inclusion Criteria

1. Signed informed consent.
2. Patients with advanced PAOD (Fontaine stage IV), evaluated by a vascular surgeon prior to inclusion. Diagnosis was based on ankle-brachial index (ABI) < 0.90; in patients with ABI > 1.40, duplex Doppler ultrasound demonstrating monophasic distal flow confirmed the diagnosis [13].

**Figure 1:**
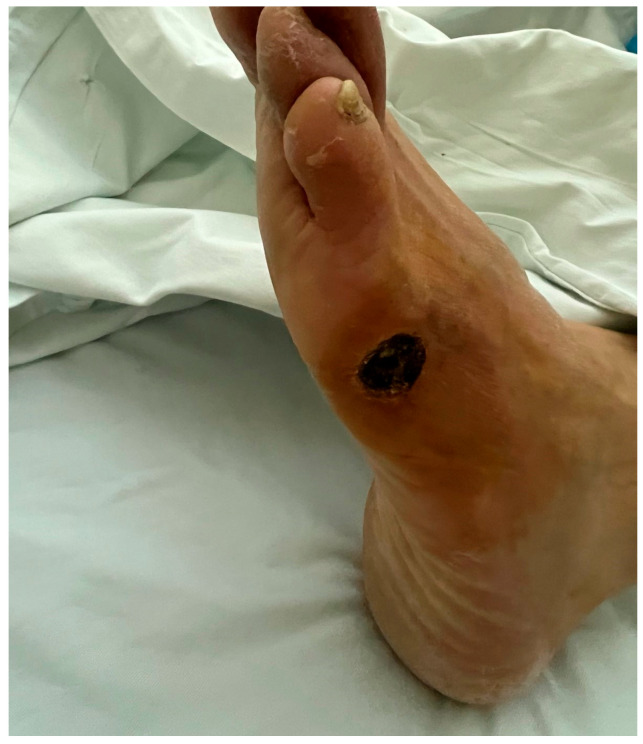
Representative clinical image of advanced peripheral arterial occlusive disease in one of the included patients.

All patients had ischemic ulceration considered clinically consistent with advanced PAOD. Ulcer size (cm^2^) was measured digitally at baseline and at the end of the 4-week observation period, and ulcer duration was recorded. In diabetic patients, a neuroischemic component could not be completely excluded; however, all ulcers were clinically adjudicated by a vascular surgeon as primarily ischemic in the context of advanced PAOD. A representative clinical image of one of the included patients with advanced peripheral arterial occlusive disease is presented in Figure 1. Ulcer size was included as a descriptive clinical variable to characterize wound burden and assess comparability between groups, but it was not predefined as a primary or secondary efficacy endpoint because the present study was designed to evaluate platelet reactivity over a short observation period rather than wound-healing outcomes. Patients received standard medical therapy, including antiplatelet treatment. Prior revascularization status was recorded as open surgery, endovascular treatment, or no option for revascularization. No open or endovascular revascularization procedures were performed during the 4-week observation period. Background medical therapy and wound care were maintained according to routine vascular care, with no systematic between-group differences in treatment policy during the 4-week observation period. Systemic inflammation was assessed using CRP and PLR.

### 2.3. Exclusion Criteria

Patients younger than 18 years, pregnant patients, patients unable to provide a reliable history, and those with non-arterial ulcers, severe chronic obstructive pulmonary disease (COPD), Global Initiative for Chronic Obstructive Lung Disease stage 4 (GOLD 4) recent chemotherapy, immunosuppression or corticosteroid therapy, metastatic malignancy, left ventricular ejection fraction (LVEF)< 30%, recent thoracic or middle-ear surgery, or uncontrolled epilepsy were excluded. Patients with gangrene were also excluded.

### 2.4. Baseline Assessment

Baseline evaluation included clinical examinations, structured interviews, and venous blood sampling. Data collected included demographics, cardiovascular risk factors, smoking status, comorbidities, prior revascularization, and medication use, particularly antiplatelet therapy and statins. Patients receiving acetylsalicylic acid, clopidogrel, their combination, or no antiplatelet therapy were included; other antiplatelet agents were excluded.

### 2.5. Sample Size Calculation

In the absence of prior directly comparable HBOT studies in PAOD using Multiplate outcomes, sample size estimation was based on the closest available published interventional data evaluating changes in platelet aggregation after HBOT in patients with diabetes mellitus, with G*Power 3.0 (α = 0.05, β = 0.20, 2:1 allocation), requiring at least 60 HBOT and 30 control patients [14,15].

### 2.6. Study Groups

Participants were allocated to the HBOT group (*n* = 60) or waiting-list control group (*n* = 30) according to treatment availability. Control patients continued to receive standard care without HBOT during the 4-week observation period. Blood sampling and Multiplate testing were performed at identical time points in both groups in order to minimize time-related variability, and baseline characteristics were compared between groups.

### 2.7. Measured Variables

The following variables were assessed at baseline and after 4 weeks: ABI (MESI ABPI MD), complete blood count (CBC), CRP, lipid profile, urea, creatinine, eGFR, platelet aggregation, PLR and ulcer size. Pain intensity was assessed using a visual analogue scale (VAS) at baseline and at the 4-week follow-up visit. Antiplatelet therapy was considered a potential confounder.

### 2.8. Hyperbaric Oxygen Therapy Protocol

HBOT was administered in 20 sessions over 4 weeks (5 sessions/week), at 2.0–2.5 atmospheres absolute (ATA) for 60 min [16], using single-place chambers (BaroxSolo; Yaklaşım Makine/BaroxHBO, Istanbul, Turkey).

### 2.9. Platelet Aggregation Testing

Platelet aggregation was measured using Multiplate^®^ impedance aggregometry (Roche Diagnostics) according to the manufacturer’s instructions. Whole blood was collected in lithium-heparin tubes and analyzed within the recommended timeframe. Aggregation was assessed using ASPI, ADP, and TRAP agonists and expressed as AUC. Previous comparative studies reported no significant differences in Multiplate AUC between heparin- and hirudin-anticoagulated samples [17].

Standardized sampling, anticoagulant use, and analytical conditions were applied across all samples. Blood was collected by venipuncture, gently inverted, allowed to rest under standardized conditions, and analyzed 30 min after collection. All analyses were performed at the Department of Transfusion Medicine, University Clinical Center Kragujevac.

### 2.10. Hematologic and Biochemical Analyses

CBC was measured using an automated hematology analyzer (Beckman Coulter DxH 900, Beckman Coulter, Brea, CA, USA). Biochemical parameters (CRP, lipids, urea, creatinine, eGFR) were analyzed from serum using an automated analyzer (Abbott Alinity c, Abbot, Chicago, IL, USA). All analyses were performed at the Department of Laboratory Diagnostics.

### 2.11. Statistical Analyses

Statistical analysis was performed using IBM SPSS Statistics 23. Data were presented as mean ± SD or as frequencies, as appropriate. Distribution was assessed by the Kolmogorov–Smirnov test. Within-group changes were analyzed using the paired *t*-test or Wilcoxon signed-rank test, and between-group differences using the independent t-test or Mann–Whitney U test, as appropriate.

To account for repeated within-subject measurements and to estimate population-averaged associations over time, generalized estimating equation (GEE) models with an identity link were constructed for ASPI-, ADP-, and TRAP-induced aggregation. β coefficients represent absolute differences in platelet aggregation (AUC). Adjusted models included clinically relevant confounders known to influence platelet reactivity, namely antiplatelet therapy, diabetes mellitus, smoking status, and CRP. A parsimonious modeling strategy was chosen because of the sample size and the repeated-measures design. A *p*-value < 0.05 was considered statistically significant.

During the preparation of this work, the authors used generative (AI) assisted tools for text shortening and English language editing. After using these tools, the authors reviewed and edited the content as needed and take full responsibility for the content of the published article.

## 3. Results

**Figure 2:**
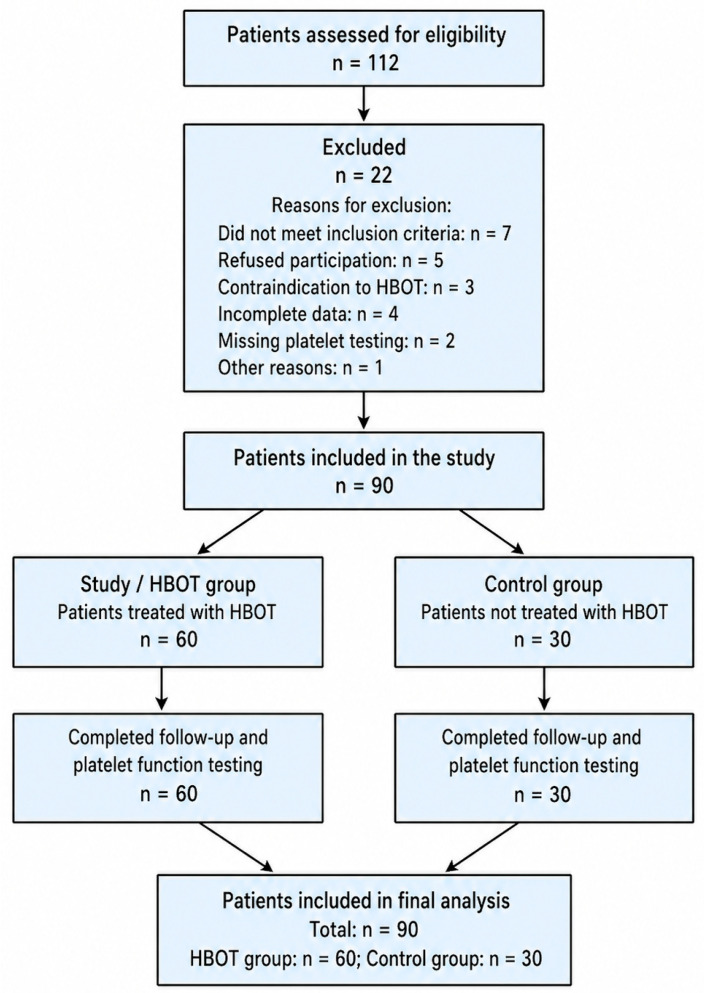
Flowchart of patient inclusion and exlusion, HBOT, hyperbaric oxygen therapy.

A total of 90 patients were included in the study, with 60 in the HBOT group and 30 in the control group. The mean age of participants was 66.8 ± 9.4 years (range 37–85), and 63 patients (70%) were male. Diabetes mellitus was present in 57.8% of the cohort. Overall, the two groups were broadly comparable at baseline with respect to the main demographic and clinical characteristics. Figure 2 shows the flowchart of patient inclusion and exclusion. A total of 112 patients were assessed for eligibility, of whom 22 were excluded for predefined reasons. The final study population included 90 patients, with 60 patients in the HBOT group and 30 patients in the control group, all of whom completed follow-up and platelet function testing.

Statin use was similar between groups. Seven patients in the HBOT group and four in the control group (11.7% and 13.3%, respectively) had an ankle–brachial index (ABI) > 1.40, suggesting non-compressible arteries. Prior revascularization status was also documented in both groups, including patients with previous open surgery, previous endovascular treatment, and patients without an option for revascularization. Importantly, baseline ulcer size did not differ statistically significantly between the HBOT and control groups (Table 1).

After 4 weeks, platelet aggregation measured by the Multiplate analyzer (ASPI, ADP, and TRAP) was reduced in the HBOT group, whereas no meaningful change was observed in the control group (Table 2).

In adjusted GEE analyses that included antiplatelet therapy (acetylsalicylic acid, clopidogrel, or their combination), diabetes mellitus, smoking status, and C-reactive protein, a statistically significant group × time interaction was observed for all three platelet activation pathways, indicating that the change over time differed significantly between the HBOT and control groups.

For ASPI-induced aggregation, GEE analysis demonstrated a significant group × time interaction (β = −290.5; 95% CI −374.5 to −206.4; *p* < 0.001), indicating a greater reduction over time in the HBOT group than in the control group. Estimated marginal means showed a marked decline in the HBOT group, whereas no significant change was observed in controls. These findings indicate that HBOT was associated with a greater reduction in ASPI values compared with standard care alone, independently of the covariates included in the model (Table 3).

Similarly, GEE analysis for ADP-induced aggregation demonstrated a significant group × time interaction (β = −243.6; 95% CI −316.97 to −170.2; *p* < 0.001), with a marked reduction in the HBOT group and no significant change in controls. HBOT was therefore associated with a greater reduction in ADP-induced platelet aggregation, independently of antiplatelet therapy, diabetes, smoking status, and CRP levels (Table 4).

For TRAP-induced aggregation, GEE analysis showed a significant group × time interaction (β = −330.9; 95% CI −422.4 to −239.4; *p* < 0.001), again indicating a greater reduction in the HBOT group than in controls. Estimated marginal means confirmed a pronounced decrease after HBOT, while no significant change was observed in the control group. HBOT was thus associated with a robust reduction in TRAP-induced platelet aggregation compared with the control group (Table 5).

A sensitivity analysis excluding patients with ABI > 1.40 was conducted to address potential diagnostic uncertainty related to non-compressible arteries. The observed associations between HBOT and platelet aggregation remained consistent in direction and magnitude for ASPI-, ADP-, and TRAP-induced aggregation.

No increase in inflammatory markers was observed following HBOT, as reflected by stable or reduced CRP and PLR values. These findings suggest that HBOT was not associated with an increase in systemic inflammatory activity over the study period (Table 6).

**Figure 3:**
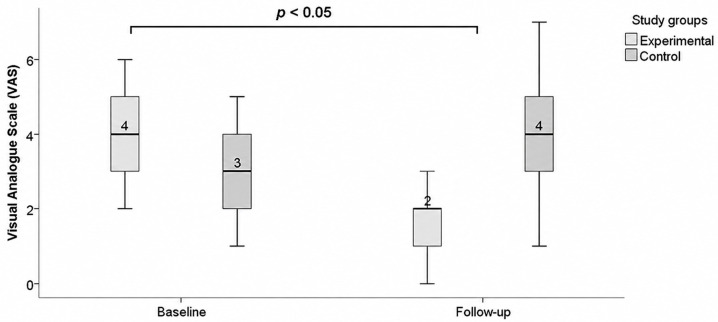
Changes in pain intensity assessed by the visual analogue scale (VAS) in the HBOT and control groups over the 4-week study period.

In the experimental group receiving HBOT, pain intensity on the visual analogue scale (VAS) decreased from a median of 4 to 2, indicating a clinically relevant reduction in perceived pain. In contrast, the control group showed a slight increase in VAS scores, from 3 to 4. These findings suggest that HBOT may be associated with a meaningful reduction in pain compared with the control group (Figure 3).

**Figure 4:**
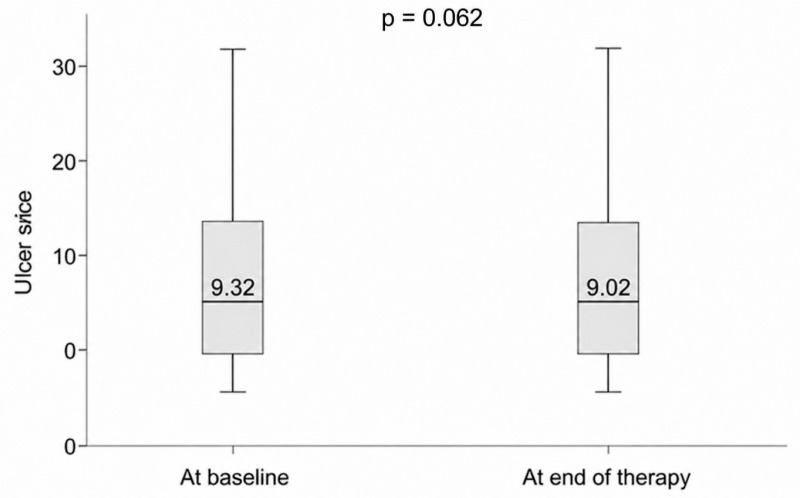
Changes in ulcer size before and after HBO therapy.

Ulcer size showed a slight numerical reduction from baseline to the end of the observation period in HBO group (9.32 vs. 9.02), but this change did not reach statistical significance (*p* = 0.062) (Figure 4).

## 4. Discussion

The present study evaluated whether hyperbaric oxygen therapy (HBOT), used as an adjunct to standard treatment, is associated with changes in platelet aggregation in patients with advanced PAOD. The main finding was that, over a 4-week period, HBOT was associated with a consistent reduction in platelet aggregation across ASPI-, ADP-, and TRAP-induced pathways, whereas no comparable change was observed in the waiting-list control group. HBOT was also associated with reduced pain intensity, while CRP and PLR remained stable throughout the observation period.

Previous studies have described effects of HBOT on platelet-to-lymphocyte ratio and inflammatory markers, supporting its potential antithrombotic and anti-inflammatory role. However, much of this evidence derives from heterogeneous populations or different clinical settings, which limits direct extrapolation to patients with PAOD [18,19]. In PAOD itself, prior work has mainly focused on platelet hyperreactivity and resistance to antiplatelet therapy [4], whereas the effects of HBOT on platelet function have not been systematically investigated. In that context, the present findings are relevant because they suggest that adjunctive HBOT may be associated with reduced platelet reactivity in a population characterized by high thrombotic burden despite standard vascular care.

In our study, the reduction in platelet aggregation was observed across three different activation pathways assessed by Multiplate (ASPI, ADP, and TRAP), which strengthens the internal consistency of the finding. Importantly, the analyses accounted for concomitant antiplatelet therapy and other clinically relevant confounders, supporting the interpretation that the observed differences were not explained solely by background treatment. Multiplate impedance aggregometry allows assessment of platelet reactivity under near-physiological conditions and correlates with light transmission aggregometry, which supports its clinical relevance [20]. It has also been used to identify residual platelet activity in patients receiving antiplatelet therapy [21], although its predictive value varies across clinical settings [22]. In the present study, its use represents a methodological strength because it enabled pathway-specific assessment of platelet function in a clinically high-risk PAOD cohort.

These findings should be interpreted in the context of previously heterogeneous data. In vitro studies have demonstrated potential pro-aggregatory effects of hyperoxia [23], whereas animal studies have reported reduced platelet aggregation following HBOT [24]. Such discrepancies likely reflect differences in experimental models, exposure protocols, and the biological context in which oxygen excess is studied. Against that background, our results are clinically informative because they derive from patients with advanced chronic arterial disease rather than from isolated experimental systems.

More recent clinical observations also support this direction of effect. A study in patients with type 2 diabetes reported decreased platelet aggregation after short-term HBOT [15], which is concordant with our findings. Although the populations are not identical, the consistency in direction strengthens the possibility that HBOT may modulate platelet reactivity in selected chronic vascular and metabolic conditions.

The mechanisms underlying this association are likely multifactorial. HBOT increases plasma oxygen levels, improves tissue oxygenation, and may improve microcirculatory flow, thereby reducing local ischemic stimuli that contribute to platelet activation [25]. HBOT also affects pathways related to vascular tone, angiogenesis, and oxidative balance [26], which may indirectly influence platelet function. While the present study was not designed to define mechanisms, these pathways provide biological plausibility for the observed reduction in platelet reactivity.

This interpretation is also consistent with broader data on HBOT in ischemic lower-extremity disease. In patients with diabetic foot and PAOD, HBOT has been associated with improved microcirculation and lower amputation rates, although effects on wound healing remain heterogeneous across studies [27]. HBOT has also been linked to reduced oxidative stress and inflammatory cytokines and to promotion of angiogenesis [28]. Favorable effects on hemorheology, including improved erythrocyte deformability, have been described as well [29], although neutral findings have also been reported [30]. Taken together, these observations support the view that HBOT does not adversely affect, and may improve, the microcirculatory environment in which platelet activation occurs.

This point is clinically relevant because patients with PAOD are characterized by increased platelet activation and a persistent prothrombotic state [31], while residual platelet reactivity despite antiplatelet therapy has been associated with adverse outcomes [32]. Within that framework, the present findings suggest that HBOT may have effects extending beyond tissue oxygen delivery alone and may be associated with measurable changes in thrombosis-related pathways. At the same time, these laboratory findings should not be interpreted as proof of clinical benefit, since outcomes such as ulcer healing, limb salvage, amputation, and cardiovascular events were not predefined efficacy endpoints in the present study.

In addition to the laboratory findings, HBOT was associated with reduced pain intensity over the study period. This observation is in line with previous reports describing analgesic effects of HBOT [33]. The most plausible explanation in the present setting is improved tissue oxygenation together with modulation of inflammatory and ischemia-related pathways. Evidence from other chronic pain-related conditions, including post-COVID syndrome and fibromyalgia, suggests that HBOT may also influence pain processing and neuroinflammatory mechanisms [34,35]. Although these data come from different clinical contexts, they support the biological plausibility of the reduction in pain observed in our PAOD cohort.

Regarding inflammation, no increase in CRP or PLR was observed during follow-up, suggesting that HBOT was not associated with an overt systemic pro-inflammatory response in this cohort [36]. This is consistent with studies showing predominantly immunomodulatory effects of HBOT, including reductions in inflammatory markers across different conditions [37,38,39,40]. Although transient cytokine changes have been reported in some settings [41], the overall pattern in our study does not support a clinically relevant pro-inflammatory effect during the observed treatment period.

Although hirudin is commonly preferred for Multiplate testing, lithium heparin was used consistently under a standardized preanalytical protocol in all participants. Because sample collection, handling, timing, anticoagulant use, and analytical conditions were identical across groups, this factor is unlikely to explain the between-group differences observed over time. Previous comparative studies have also reported no significant differences in Multiplate AUC between heparin- and hirudin-anticoagulated samples [17]. Nevertheless, a modest methodological influence of anticoagulant choice cannot be completely excluded.

Recent advances in the treatment of peripheral arterial disease have expanded the role of revascularization, particularly in patients with severe symptoms, non-healing ulcers, or chronic limb-threatening ischemia. Endovascular procedures, such as balloon angioplasty with or without stent implantation, have become widely used because they are minimally invasive and can improve blood flow, relieve ischemic symptoms, and support wound healing. Drug-coated balloons and drug-eluting stents may further reduce restenosis in selected patients. Nevertheless, open surgical bypass remains an important treatment option, especially in patients with extensive or complex arterial lesions, long occlusions, or when endovascular therapy is not technically suitable or has failed. Therefore, the choice between endovascular treatment, stenting, open surgery, or hybrid revascularization should be individualized according to lesion anatomy, disease severity, comorbidities, and expected long-term benefit [32,42].

Autoamputation may occur in cases of dry ischemic gangrene, when necrotic tissue becomes clearly demarcated and separates spontaneously. However, waiting for autoamputation in non-operative management may be associated with prolonged healing, discomfort, risk of infection, reduced quality of life, and delayed definitive treatment. Therefore, this approach should be considered only in carefully selected patients who are not suitable candidates for revascularization or surgical amputation, with close clinical monitoring. In most patients with chronic limb-threatening ischemia, early vascular assessment and timely revascularization or surgical management remain preferred when feasible [43,44].

Although vasopressor-induced acral ischemia and necrosis are not directly related to peripheral arterial occlusive disease, they represent an important differential consideration in critically ill patients. Unlike PAOD, which is mainly caused by chronic atherosclerotic arterial obstruction, vasopressor-related ischemia is usually associated with intense peripheral vasoconstriction, microcirculatory impairment, shock-related hypoperfusion, and prothrombotic changes. This complication is most often described in patients requiring high-dose or prolonged vasopressor therapy during septic or cardiogenic shock and may lead to digital or limb necrosis even in the absence of major arterial occlusion. Therefore, careful monitoring of distal perfusion, early recognition of ischemic changes, and timely vascular or surgical consultation are important to reduce tissue loss and improve clinical outcomes [45,46].

Baseline ulcer size was comparable between groups, and no statistically significant change in ulcer size was observed over the 4-week study period. This finding is important for interpretation because it indicates that the observed changes in platelet aggregation were not accompanied by measurable short-term changes in wound size within the same interval. At the same time, ulcer size was not a predefined efficacy endpoint, and the follow-up period was relatively short. Accordingly, these findings should not be interpreted as evidence against a possible longer-term effect of HBOT on wound healing.

The findings should also be considered in light of the study design. This was a prospective observational study, and allocation to HBOT or control was based on treatment availability within routine care rather than randomization. Although the groups were broadly comparable at baseline and the analyses were adjusted for major confounders, residual confounding cannot be fully excluded. In addition, platelet reactivity was assessed over a relatively short follow-up period, so the persistence of the observed effect remains uncertain.

Overall, the observed reduction in platelet aggregation across multiple activation pathways, together with lower pain intensity and stable inflammatory markers, supports the possibility that adjunctive HBOT may influence thrombotic and microcirculatory processes in patients with advanced PAOD. These findings add to the limited data on the relationship between HBOT and platelet function in this population and provide a rationale for further studies focused on durability of effect and clinically meaningful vascular outcomes.

### Limitations of the Study

This study has several limitations. It was a single-center observational study with a relatively small sample size, and treatment allocation was determined by HBOT availability in routine clinical practice rather than by randomization. Although the groups were broadly comparable at baseline and the analyses were adjusted for major confounders, residual confounding cannot be fully excluded. Platelet function was assessed using a single method, multiplate impedance aggregometry, which captures selected activation pathways but does not provide a comprehensive assessment of all aspects of platelet biology. The follow-up period was relatively short, and the durability of the observed changes remains unknown. In addition, the study was not designed or powered to assess hard clinical outcomes such as ulcer healing, limb salvage, amputation, or cardiovascular events. These limitations should be considered when interpreting the findings.

## 5. Conclusions

In this prospective observational study, adjunctive HBOT was associated with reduced platelet reactivity across multiple activation pathways in patients with advanced PAOD. HBOT was also associated with lower pain intensity, without an accompanying increase in CRP or PLR over the study period. These findings suggest that HBOT may influence thrombotic and microcirculatory pathways in this clinical setting. Further studies are needed to determine the durability of these effects and their clinical relevance.

## Figures and Tables

**Table 1:** Basic characteristics of the study population.

Variable	Category	Experimental Group		Control Group	
		N	%	N	%
Sex	Male	41	68.3%	22	73.3%
	Female	19	31.7%	8	26.7%
Age	Mean ± SD	66.82 ± 9.42		63 ± 8.31	
Diabetes mellitus	Yes	33	55.0%	19	63.3%
	No	27	45.0%	11	36.7%
Antiplatelet therapy	None	18	30.0%	9	30.0%
	Acetylsalicylic acid	28	46.7%	10	33.3%
	Clopidogrel	4	6.7%	-	-
	Aspirin + clopidogrel	10	16.7%	11	36.7%
Ulcer size (cm^2^)	Mean ± SD	9.32 ± 7.22		9.81 ± 7.85	
Ulcer duration (months)	Mean ± SD	11.15 ± 12.37		10.00 ± 6.37	
EGF	Mean ± SD	91.45 ± 28.98		77.23 ± 25.89	
Use of statins	Yes	31	51.7%	16	53.3%
	No	29	48.3%	14	46.7%
PCT	Mean ± SD	0.24 ± 0.08		0.25 ± 0.08	
Prior revascularisation	Open surgery	20	33.3%	9	30.0%
	Endovascular	22	36.7%	13	43.3%
	No option for revascularisation	18	30.0%	8	26.7%
ABI	Below 0.9	53	88.3%	26	86.7%
	Above 1.4	7	11.7%	4	13.3%
BMI	Mean ± SD	28.23 ± 4.51		26.57 ± 2.93	

**Table 2:** Platelet aggregation values (ASPI, ADP, and TRAP) at baseline and after 4 weeks.

Test (AUC)	Group	Baseline Mean ± SD	Follow-Up Mean ± SD
ASPI	Control	712.70 ± 293.75	725.05 ± 272.81
	HBOT	756.25 ± 309.47	466.11 ± 300.07
ADP	Control	614.73 ± 424.06	606.15 ± 412.11
	HBOT	706.06 ± 437.57	462.81 ± 441.74
TRAP	Control	1066.92 ± 349.63	1098.98 ± 355.65
	HBOT	1023.95 ± 355.69	693.10 ± 351.72

Abbreviations: HBOT, hyperbaric oxygen therapy; ASPI, arachidonic-acid-induced aggregation; ADP, adenosine-diphosphate-induced aggregation; TRAP, thrombin-receptor-activating peptide-induced aggregation; AUC, area under the curve.

**Table 3:** Generalized estimating equation (GEE) analysis of ASPI-induced platelet aggregation.

Parameter	B	Std. Error	95% Wald Confidence Interval		Hypothesis Test		
			Lower	Upper	Wald Chi-Square	df	Sig.
(Intercept)	375.269	58.0817	261.431	489.107	41.745	1	0.000
[Group = 0]	246.942	66.1935	117.205	376.679	13.917	1	0.000
[Time = 0]	290.141	42.8676	206.122	374.160	45.810	1	0.000
[Therapy = 0]	45.361	63.4430	−78.985	169.707	0.511	1	0.475
[Diabetes = 0]	9324	61.2597	−110.743	129.391	0.023	1	0.879
[Smoking = 0]	112.732	57.1970	0.628	224.836	3885	1	0.049
CRP	0.765	2.3316	−3805	5335	0.108	1	0.743
[Group = 0] × [time = 0]	−290.498	42.8836	−374.549	−206.448	45,889	1	0.000
(Scale)	104,448,699						

**Table 4:** Generalized estimating equation (GEE) analysis of ADP-induced platelet aggregation.

Parameter	B	Std. Error	95% Wald Confidence Interval		Hypothesis Test		
			Lower	Upper	Wald Chi-Square	df	Sig.
(Intercept)	380.931	79.5277	225.060	536.802	22,943	1	0.000
[Group = 0]	152.250	70.9392	13.212	291.289	4606	1	0.032
[time = 0]	243.248	37.4463	169.854	316.641	42,197	1	0.000
[Therapy = 0]	−50.153	67.6456	−182.736	82.430	0.550	1	0.458
[Diabetes = 0]	26.936	70.4173	−111.079	164.951	0.146	1	0.702
[Smoking = 0]	147.468	64.7337	20.592	274.343	5190	1	0.023
CRP	2117	3.3285	−4407	8641	0.405	1	0.525
[Group = 0] × [time = 0]	−243.581	37.4437	−316.969	−170.192	42.318	1	0.000
(Scale)	107,990,239						

**Table 5:** Generalized estimating equation (GEE) analysis of TRAP-induced platelet aggregation.

Parameter	B	Std. Error	95% Wald Confidence Interval		Hypothesis Test		
			Lower	Upper	Wald Chi-Square	df	Sig.
(Intercept)	609.319	86.7035	439.383	779.254	49.387	1	0.000
[Group]	373.882	75.2609	226.373	521.391	24.679	1	0.000
[Time]	330.852	46.6667	239.387	422.317	50.263	1	0.000
[Therapy]	−69.281	69.9007	−206.284	67.722	0.982	1	0.322
[Diabetes]	30.169	70.8256	−108.647	168.985	0.181	1	0.670
[Smoking]	168.291	69.8023	31.481	305.101	5813	1	0.016
CRP	2057	2.7018	−3238	7352	0.580	1	0.446
[Group = 0] × [time = 0]	−330,916	46.6829	−422.413	−239.419	50.248	1	0.000
(Scale)	125,129,999						

**Table 6:** The effect of HBO therapy on inflammatory markers.

	Statistical Significance Test		Median	
	z Statistics	*p*	Before HBO	After HBO
PLR—experimental	0.913	0.361	134.55	146
CRP—experimental	1.220	0.222	3.7	3.4
PLR—control	0.545	0.586	151.99	154.86
CRP—control	1.732	0.083	7.73	7.64

## Data Availability

The data presented in this study are not publicly available due to privacy and ethical restrictions related to patient-level clinical data.

## Abbreviations

The following abbreviations are used in this manuscript:

PAOD	peripheral arterial occlusive disease
HBOT	hyperbaric oxygen therapy
ASPI	arachidonic-acid-induced platelet aggregation test
ADP	adenosine diphosphate
TRAP	thrombin-receptor-activating peptide
CRP	C-reactive protein
PLR	platelet-to-lymphocyte ratio
VAS	visual analogue scale
STROBE	Strengthening the Reporting of Observational Studies in Epidemiology
ABI	ankle–brachial index
CBC	complete blood count
eGFR	estimated glomerular filtration rate
AUC	area under the curve
GEE	generalized estimating equations
COPD	chronic obstructive pulmonary disease
GOLD	Global Initiative for Chronic Obstructive Lung Disease
LVEF	left ventricular ejection fraction
ATA	atmospheres absolute
